# Respiratory Sinus Arrhythmia is Mainly Driven by Central Feedforward Mechanisms in Healthy Humans

**DOI:** 10.3389/fphys.2022.768465

**Published:** 2022-07-07

**Authors:** Maria Skytioti, Maja Elstad

**Affiliations:** Division of Physiology, Institute of Basic Medical Sciences, University of Oslo, Oslo, Norway

**Keywords:** respiratory sinus arrhythmia, cardiovascular oscillations, central respiratory drive, cardiorespiratory interactions, pulmonary stretch reflex

## Abstract

Heart rate variability (HRV) has prognostic and diagnostic potential, however, the mechanisms behind respiratory sinus arrhythmia (RSA), a main short-term HRV, are still not well understood. We investigated if the central feedforward mechanism or pulmonary stretch reflex contributed most to RSA in healthy humans. Ventilatory support reduces the centrally mediated respiratory effort but remains the inspiratory stretch of the pulmonary receptors. We aimed to quantify the difference in RSA between spontaneous breathing and ventilatory support. Nineteen healthy, young subjects underwent spontaneous breathing and non-invasive intermittent positive pressure ventilation (NIV) while we recorded heart rate (HR, from ECG), mean arterial pressure (MAP) and stroke volume (SV) estimated from the non-invasive finger arterial pressure curve, end-tidal CO_2_ (capnograph), and respiratory frequency (RF) with a stretch band. Variability was quantified by an integral between 0.15–0.4 Hz calculated from the power spectra. Median and 95% confidence intervals (95%CI) were calculated as Hodges–Lehmann’s one-sample estimator. Statistical difference was calculated by the Wilcoxon matched-pairs signed-rank test. RF and end-tidal CO_2_ were unchanged by NIV. NIV reduced HR by 2 bpm, while MAP and SV were unchanged in comparison to spontaneous breathing. Variability in both HR and SV was reduced by 60% and 75%, respectively, during NIV as compared to spontaneous breathing, but their interrelationship with respiration was maintained. NIV reduced RSA through a less central respiratory drive, and pulmonary stretch reflex contributed little to RSA. RSA is mainly driven by a central feedforward mechanism in healthy humans. Peripheral reflexes may contribute as modifiers of RSA.

## Introduction

Cardiovascular oscillations such as heart rate variability (HRV) can be used as a prognostic marker for several diseases (Bigger et al., 1993; Task Force of the European Society of C and the North American Society of Pacing E, 1996). The presence of HRV may be an indication of a healthy heart. A main component of short-term HRV is respiratory sinus arrhythmia (RSA) (Skytioti et al., 2017), a well-known phenomenon describing inspiratory increases and expiratory decreases in heart rate (HR) (Ludwig, 1847). The respiratory-related changes in vagal outflow give rise to RSA, which is suggested to be elicited by several mechanisms and to have at least as many proposed functions.

Two of the suggested contributors to RSA are the central feedforward mechanism and pulmonary stretch reflexes (Anrep et al., 1936a; Anrep et al., 1936b; Freyschuss and Melcher, 1975). The debate about whether a central feedforward mechanism is the main contributor to RSA, or if peripheral reflexes such as pulmonary stretch reflexes are the most important determinant of RSA (Taha et al., 1985–1995), is by no means settled (Elstad et al., 2018). Other peripheral mechanisms such as arterial baroreflex and cardiopulmonary reflexes are also suggested to contribute (Eckberg and Karemaker, 2009).

We investigated if central feedforward mechanisms or the pulmonary stretch reflex are the most important contributor to RSA in healthy humans. Many previous studies have been performed in animals, which have many similarities to human cardiovascular control mechanisms. We wanted to develop a method that could differentiate the response between the central respiratory drive and the peripheral pulmonary stretch reflex into the amplitude of RSA.

Clinically assisted ventilation modes support respiration in patients with respiratory failure who are able to initiate inspiration themselves. In this setting, the central respiratory drive is present; therefore, RSA amplitude is maintained (Cooper et al., 2004). However, when the respiratory drive was diminished by hypocapnia, RSA amplitude was significantly reduced (Cooper et al., 2004). In our study, we thus employed a control-mode non-invasive intermittent positive pressure ventilation (NIV) in order to suppress the inspiratory drive with a normal lung inflation-deflation cycle to maintain the pulmonary stretch reflex.

In this study, we reanalyzed data from a previous study to investigate if we could separate the central control mechanisms from pulmonary stretch reflexes on RSA in healthy humans. We assumed that the experimental protocol with NIV would decrease RSA substantially. Our hypothesis was that RSA is mainly driven by central feedforward control mechanisms. If our hypothesis was confirmed, RSA would decrease during NIV. We investigated other cardiovascular variables known to influence RSA as well.

## Methods

### Subjects

We recruited 22 subjects. Three did not complete the protocol due to difficulties accepting the NIV protocol (two) or technical problems with the blood pressure measurements. The 19 included in the analysis had a median age of 21 years (range 19–25), a height of 174 cm, a weight of 65 kg, a body surface area of 1.8 m^2^, and performed exercise on a median of 5 h per week. Ten of the participants were female. All subjects gave written informed consent prior to the experiments. The experimental protocol was preapproved by the regional ethical committee (Ref.no: 2012/2251) and conformed with the Helsinki declaration.

Subjects abstained from strenuous exercise and alcohol for at least 24 h prior to the experiments. All subjects had a light meal for at least 2 h and avoided all caffeine-contained beverages or food for 12 h prior to the experiments. All subjects were healthy and took no medications except contraceptives.

### Experimental Protocol

Analyses from parts of these experiments have been published previously (Elstad and Walløe, 2015; Skytioti et al., 2018). Only the protocol relevant to this study is described here.

Subjects visited the lab facilities at least twice, with familiarization with the recording equipment and NIV on the first visit. During the experiments, the subject rested supine with recording equipment attached and breathing through a comfortable face mask (Respireo Primo F Non-Vented, Air Liquide Medical Systems, Italy) covering both the nose and mouth. NIV was given by VIVO50 (Diacor a/s, Oslo, Norway), a ventilator fitted for home usage, and provided intermittent positive pressure at an individually adjusted frequency. We adjusted individually the inspiration time [median 1.5 s (range 1.2–1.8 s)], inspiratory pressure (minimum: median 6 cm H_2_O, maximum: median 15 cm H_2_O), and low expiratory pressure (range 2–3 cm H_2_O).

The individual subject’s spontaneous breathing frequency was determined on the experimental day. The subject breathed spontaneously for a minimum of two minutes unaware of the observation of their respiration. The recorded respiratory trace was inspected for stability, and respiratory frequency was estimated in breaths per min. The breathing rate was adjusted upward to suppress the initiation of inspiration. The ventilator’s frequency was then set to the subject’s individual respiratory frequency.

We employed control-mode NIV. The subjects were trained not to initiate inspiration themselves but to passively accept the tidal volume and breathing frequency given by the ventilator. This experimental method has reduced RSA substantially compared to the physiologic setting of spontaneous breathing due to the reduction in the central feedforward drive and the elimination of the spontaneous inspiratory effort (Beda et al., 2012; Elstad et al., 2015; Skytioti et al., 2017).

### Instrumentation and Recordings

Respiratory chest movement (RE) was obtained using a belt around the upper abdomen (Respiration and Body Position Amplifier, Scan-Med a/s, Drammen, Norway). HR was obtained from the duration of each RR interval of the three-lead ECG signal (SD-100, Vingmed Sound, Horten, Norway). The recording computer has a customized R detector. Finger arterial pressure was recorded continuously from the middle-left finger positioned at heart level (Finometer, Finapres Medical System, Amsterdam, Netherlands). Beat-by-beat mean arterial blood pressure (MAP) was calculated by numerical integration in each RR interval by the recording computer. Cardiac stroke volume (SV) was calculated by the incorporated Modelflow (Bogert and van Lieshout, 2005). During supine rest, SV measured by Modelflow is found to be in good accordance with SV measured by ultrasound Doppler (Van Lieshout et al., 2003). Cardiac output (CO) was calculated from the corresponding pulse rate and SV estimated by the Finometer. The signals were sampled at 100 Hz and transferred online to a recording computer running a dedicated data collection and analysis program (program for real-time data acquisition: Morten Eriksen, Oslo, Norway). A capnograph (inbuilt *in vivo*50) registered the expiratory CO_2_ level and indicated if a subject was hypoventilated or hyperventilated.

### Signal Processing, Analysis, and Statistical Tests

Every recorded signal from each experimental run was visually inspected, and only time intervals with successful recordings with stationarity were included in the subsequent analysis. Each selected continuous sequence with acceptable measurements had to last for at least ten respiratory cycles (Task Force of the European Society of C and the North American Society of Pacing E, 1996). The original recording was sampled at 300 Hz for ECG, 100 Hz for respiratory movements and SV, and beat-by-beat for HR and MAP.

**FIGURE 1:**
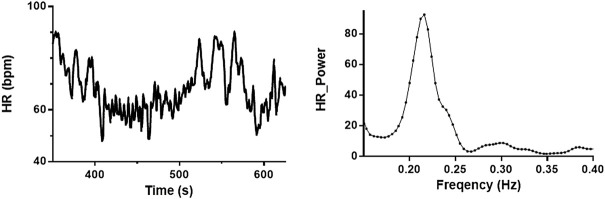
Baseline recording of heart rate and power spectrum of high-frequency interval. Heart rate (HR) recording from one subject during spontaneous breathing (left) and power spectra from the same recording (right). Respiratory sinus arrhythmia (RSA) is estimated as the area under the curve of the power spectra (0.15–0.4 Hz).

All subjects underwent 5 min of spontaneous breathing and 5 min of NIV in a randomized order. Each participant contributed in 1-3 experimental runs, and the median value of the response or the difference between the conditions is reported as one value per participant. RSA was quantified as the area under the curve within the high-frequency (HF, 0.15–0.4 Hz) interval of the power spectrum of HR (REGIST3, a program for real-time data acquisition: Morten Eriksen, Oslo, Norway, Figure 1). Power density spectra were calculated by the fast Fourier transform algorithm for each of the variables in the separate time intervals to obtain variability at 0.15–0.4 Hz (Task Force of the European Society of C and the North American Society of Pacing E, 1996). Beat-to-beat signals (HR and MAP) were by interpolation converted into equidistant time samples, resulting in 2^n^ samples as required for subsequent analysis. SV and respiratory movements already had equidistant time samples. The spectra were smoothed by a sliding Gaussian function with a standard deviation of 0.01 Hz. The other variability was calculated similarly as RSA, as previously published (Elstad, 2012; Elstad et al., 2015; Skytioti et al., 2017).

Peak respiratory frequency (RF) was determined from the power spectra of the respiration signal. During analyses, we estimated RF individually in each condition to test for consistency between spontaneous breathing and NIV. The analyzed frequency in each condition gave rise to individually assessed RF ± 0.03 Hz band. The interaction between chosen respiratory and cardiovascular variable pairs, RE-HR, MAP-HR, and HR-SV, was examined by computing coherence and phase angle from the cross-spectra at the peak RF ± 0.03 Hz. The coherence provides a measure of coupling between two signals in the range of frequencies examined. Coherence from a shorter interval centered around the respiratory frequency is higher than coherence in the classical 0.15–0.4 Hz band (Skytioti et al., 2017). Phase angles were only estimated if the coherence between the variable pair was ≥0.5.

We defined before analysis that a change in RSA of less than 10% between conditions could be considered as maintained RSA. This was based on the established practice of clinically relevant SV changes (Marik et al., 2011) and to ensure that a centrally mediated RSA mechanism was of physiological relevance.

Medians and 95% confidence intervals were calculated by Hodges–Lehmann’s estimate (Hollander and Wolfe, 1999) in a statistical program (StatXact, Cytel Studio 10, Cytel Inc., Cambridge, MA, United States). Wilcoxon matched-pairs signed-rank test was performed to test for statistical difference between the two conditions (StatXact, Cytel Studio 12, Cytel Inc., Cambridge, MA, United States). The level of significance was set to *p* < 0.05.

## Results

**FIGURE 2:**
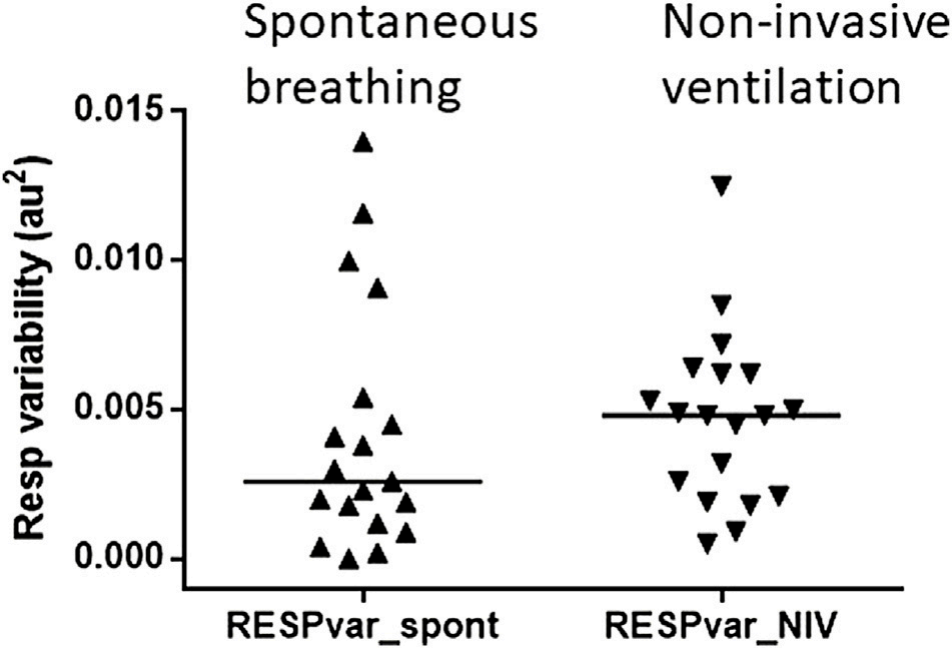
Pulmonary stretch input was maintained during non-invasive ventilation. Variability in respiratory band stretch during spontaneous breathing (left) and non-invasive ventilation (right). The triangles represent the subject in each condition. The median is indicated by the horizontal bars. The variability in the stretch of the respiratory band was similar during spontaneous breathing and non-invasive ventilation.

Nineteen volunteers completed at least one technically successful experimental protocol with 5 min of spontaneous breathing and NIV. NIV resulted in the same stretch of the respiratory band, indicating that the input to the pulmonary stretch reflex was similar in the two situations (Figure 2).

NIV showed minimal changes in the respiratory and cardiovascular variables as compared to spontaneous breathing. HR and CO decreased minimally but significantly, with 2 bpm and 0.3 l/min, respectively, from spontaneous breathing to NIV, while MAP, SV, end-tidal CO_2,_ and RF were unchanged (Table 1).

**TABLE 1:** Cardiovascular variables and respiratory frequency during spontaneous breathing and non-invasive ventilation.

Variable	Spontaneous breathing	Non-invasive ventilation
HR (bpm)	57.9 (53.0, 61.8)	55.5 ** (51.1, 60.0)
MAP (mmHg)	69.2 (64.5, 73.1)	68.8 (64.8, 72.9)
SV (ml)	89.1 (80.3, 98.1)	87.8 (80.2, 97.3)
CO (l/min)	5.18 (4.40, 5.85)	4.89* (4.20, 5.55)
RF (Hz)	0.24 (0.21, 0.28)	0.25 (0.23, 0.27)
End-tidal CO_2_ (kPa) (*n* = 10)	5.2 (4.7, 5.8)	4.9 (4.7, 5.3)

N = 19, calculated as median (95% confidence interval) by Hodges–Lehmann’s one-sample estimator. HR, heart rate; MAP, mean arterial pressure; SV, stroke volume; CO, cardiac output; RF, respiratory frequency. Bold font indicates a statistically significant change in the variable from spontaneous breathing to non-invasive ventilation. * indicates *p* < 0.05 and ** indicates *p* < 0.0001.

**FIGURE 3:**
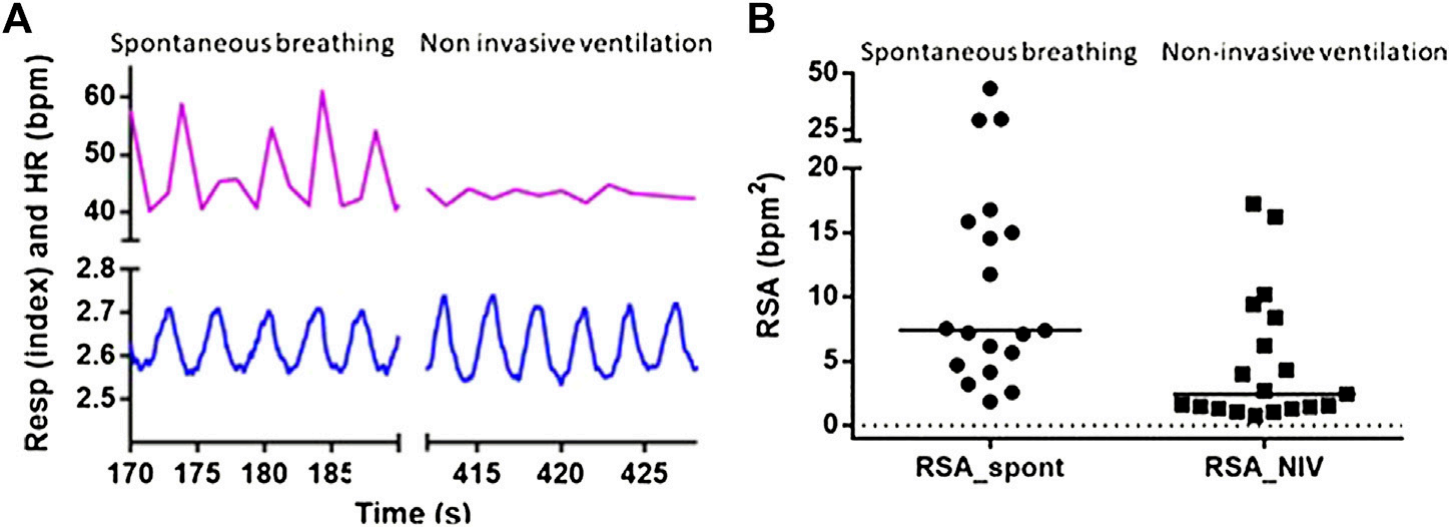
Respiratory sinus arrhythmia was decreased by non-invasive ventilation. **(A)** Five respiratory cycles during spontaneous breathing and non-invasive ventilation from one subject. Recordings of respiratory stretch (bottom) and heart rate (HR, top) show that pulmonary stretch is maintained breath-by-breath during non-invasive ventilation, while respiratory sinus arrhythmia is minimal during non-invasive ventilation. **(B)** Respiratory sinus arrhythmia is decreased for all nineteen subjects. One circle represents one subject and lines indicate the individual’s decrease in heart rate variability (HRV) from spontaneous breathing (SB) to non-invasive ventilation (NIV).

NIV reduced HRV and SVV by 60% and 75%, respectively, while MAPV and COV were unchanged as compared to spontaneous breathing (Table 2). All 19 subjects experienced a reduction in HRV, but for three of the subjects, the decrease was minimal during NIV as compared to spontaneous breathing (Figure 3). The decrease in HRV from spontaneous breathing to NIV was not significantly related to the decrease in SVV. There was also no significant change in the variability of respiration band stretch from spontaneous breathing to NIV.

**TABLE 2:** Cardiovascular variability at high-frequency interval and coherences and phases at respiratory frequency.

Variability (0.15, 0.40 Hz)	Spontaneous breathing	Non-invasive ventilation
HRV (bpm^2^)	10.2 (6.1, 16.9)	3.9 ** (1.6, 7.3)
MAPV (mmHg^2^)	2.13 (1.56, 3.24)	1.98 (1.61, 2.54)
SVV (ml^2^)	16.0 (11.9, 22.4)	4.3 ** (3.2, 7.8)
COV ((l/min)^2^)	0.03 (0.02, 0.05)	0.02 (0.015, 0.035)
Coherences (C) and phase angles (P) (radians) (RF ± 0.03 Hz)		
RE-HR	C: 0.91 (0.85, 0.94)	C: 0.81 * (0.74, 0.87)
	P: 0.28 (−0.5, −0.13)	P: 0.28 (−0.74, 0.0)
MAP-HR	C: 0.33 (0.25, 0.42)	C: 0.47 * (0.39, 0.55)
	P: N/A	P: N/A
HR-SV	C: 0.91 (0.86, 0.95)	C: 0.76 * (0.63, 0.84)
	P: 2.83 (2.62, 3.11)	P: 2.77 (2.13, 3.5)

N = 19, calculated as median (95% confidence interval) by Hodges–Lehmann’s one-sample estimator for spontaneous breathing and non-invasive ventilation; HRV, heart rate variability; MAPV, mean arterial pressure variability; SVV, stroke volume variability; COV, cardiac output variability; MAP, mean arterial pressure; HR, heart rate; RE, respiration; SV, stroke volume. Bold font indicates a statistically significant change in the variable from spontaneous breathing to NIV. * indicates *p* < 0.05 and ** indicates *p* < 0.0001. N/A means that phase angles are not estimated as coherence is below 0.5.

RE and HR showed high coherence and in-phase relationship, while HR and SV showed high coherence and inverse phase relationship during both spontaneous breathing and NIV. Coherence between MAP and HR was low during spontaneous breathing. The coherence between the pairs (RE-HR) and (HR-SV) decreased, while the coherence between MAP and HR increased during NIV as compared to spontaneous breathing (Table 2). The phase angles between the pairs did not change between the two conditions.

## Discussion

We investigated if RSA is mainly driven by central respiratory feedforward mechanisms or pulmonary stretch reflex. We reduced the central respiratory drive by exposing the subjects to NIV, which, on the other hand, maintained the cyclical pulmonary stretch. We found that the central feedforward mechanism was the main contributor to RSA in healthy humans, as NIV reduced RSA by 60%. Several mechanisms have been suggested to contribute to RSA and RSA is proposed to have several functions (Hayano et al., 1996; Sasano et al., 2002; Giardino et al., 2003; Ben-Tal et al., 2012; Elstad et al., 2015).

### Central Respiratory Drive During Non-invasive Ventilation

The three-phase respiratory motor pattern is driven by balancing excitation and inhibition within the ventrolateral respiratory column and dorsal and pontine respiratory groups (Dhingra et al., 2019). RSA is generated by the oscillation of firing in cardiac vagal motor neurons, which again are inhibited during inspiration and excited post inspiration (McAllen and Spyer, 1978; Farmer et al., 2016) (Baekey et al., 2008). Thus, RSA exhibits a classical increase in HR during the inspiratory phase, with a minimal HR during expiration (Cooke et al., 2006).

With our NIV method, we aimed at minimizing the central respiratory drive during inspiration with maintained (or increased) input from the pulmonary stretch receptors. Our subjects were trained to accept the NIV machine to initiate their breathing. The respiratory frequency of NIV matched the subject’s individual spontaneous breathing frequency to avoid effects from intrinsic oscillation. During NIV, RSA amplitude was decreased in our present study. We interpret this at our method, which overrides the subject’s central inspiratory drive and also reduced the cardiac vagal motor neurons' inspiratory inhibition and post-inspiratory excitation, leaving less RSA.

NIV can also support inspiration, with positive pressure initiated by the subject’s initiation of inspiration. If NIV was used in this manner, there would be maintained central inspiratory drive combined with increased pulmonary stretch, with maintained RSA as a result (Cooper et al., 2004).

There are several different methods to reduce RSA in healthy humans, with different potentials and limitations. While choosing the method for this study, we aimed for a method that was acceptable for the healthy subjects, non-invasive, repeatable, and available in the non-clinical laboratory. As RSA is driven by variation in vagal activity, a commonly used modifier of RSA is blockade of the vagal outflow through drugs (Toska and Eriksen, 1993; Elstad et al., 2001; Ogoh et al., 2005). Due to the pharmacological half-life of many drugs, some of these drugs need continuous infusion with clinical observation of their effect to ensure stable conditions. A common side effect is also a large increase in HR with circulatory effects. Another semi-invasive method of modifying RSA is to elevate HR artificially by electrical pacing (Hayano et al., 1996; Taylor and Eckberg, 1996; Sin et al., 2010). This also increases HR and affects circulatory regulation mechanisms such as the cardiac baroreflex. None of these methods will explore the central feedforward mechanism behind RSA.

### Pulmonary Stretch Reflex and Other Peripheral Reflexes as Input to Respiratory Sinus Arrhythmia

In our study, we maintained the stretch of pulmonary receptors during the respiratory cycle with NIV, but RSA was significantly reduced. We interpreted this finding as the central respiratory drive is the main contributor to RSA. However, in a minority of the subjects (three out of 19), we observed less than a 10% decrease in RSA, suggesting that in a subset, pulmonary stretch reflex may contribute substantially to RSA or at least maintain RSA. We aimed at assuring that NIV elicited a physiological lung stretch in the subjects, so we do not know the consequence of excessive lung stretch on the amplitude of RSA (Schelegle and Green, 2001). We also observed that end-tidal CO_2_ was unchanged. The subjects were trained on the use of NIV several times before the experiment. Two subjects were not able to accept NIV, and one difficulty was accepting the NIV breathing frequency. The included subjects all participated in the experimental protocol without discomfort or indications of increased tidal volumes.

In this study, there was no change in either MAP or MAP variability. We thus propose that the arterial baroreflex had no change in input and did not contribute to our results. Porta et al. have found that respiration affects the cardiac baroreflex pathway by reduction of baroreflex sensitivity (Porta et al., 2000; Porta et al., 2012; Porta et al., 2022). Similarly, when baroreceptor stimuli occur during inspiration, the respiratory gate renders the vagal cardiac motoneuron unresponsive (Eckberg, 2003). Our finding that RSA is mainly driven by central respiratory control has an impact on the estimation of cardiac baroreflex sensitivity. When cardiac baroreflex sensitivity is estimated from spontaneous oscillations by spectral analysis without accounting for the central feedforward component of RSA, the baroreflex sensitivity may be overestimated. This point needs further investigation in coming studies. On the other side, in our study, SVV was also reduced by NIV similar to HRV. Since the arterial baroreflexes may respond to changes in stretch produced by stroke volume (Charkoudian et al., 2005), SVV may have influenced a respiratory-related signal in the arterial baroreceptors, which again may have affected our results. Similarly, cardiopulmonary reflexes respond to changes in volume and pressure in the right side of the heart and pulmonary circulation. We cannot rule out that cardiopulmonary reflexes affected RSA in our study.

### Limitations

The subjects’ ability to tolerate NIV while awake may have influenced our results. We found that three out of 19 had maintained RSA (<10% decrease in RSA) during NIV, indicating that in a small proportion of subjects, pulmonary stretch reflex maintained RSA. Anecdotally, one of the subjects with maintained RSA was the only one with an inverse phase angle between respiration and HR changes (Freyschuss and Melcher, 1975) and may indicate a different RSA generation in that subject. Another possibility is that the subjects with maintained did not fully collaborate with the NIV and kept their central drive to respiration (Cooper et al., 2004). The subjects reported different experiences with NIV. The majority experienced breathing with support as comfortable, while a few of the participants reported the procedure as uncomfortable. We did not record which subjects were uncomfortable during NIV.

We reasoned that there were probably minimal tidal volume changes as there was neither a significant change in respiratory frequency nor end-tidal CO_2_. The stretch band has clear limitations when estimating changes in tidal volumes particularly if abdominal breathing is increased. Due to the same circumstances, the estimation of respiratory variability based on chest movement has several limitations. Future experimental protocols need to look into the effect of tidal volume change on RSA.

The time intervals used for the variability analysis varied in length and number of respiratory cycles contained. This may theoretically influence the result, however, each subject served as their own control, and any statistical analysis was performed on the change for that individual between conditions.

### Clinical Implications

Intermittent positive pressure ventilation is a common clinical intervention in patient groups with respiratory, circulatory, or neurological diseases. Increased knowledge of the circulatory effects of ventilation support will benefit the patient population and may help in the development of individualized interventions. Modern ventilators allow the patient to initiate their own breathing, a treatment that will allow more physiological cardiorespiratory interactions, and this might be beneficial for long-term treatments. Future research may investigate a combination of clinically assisted ventilation with tailored breathing frequency, tidal volume, and inspiratory:expiratory ratio to meet the patient’s individual needs for cardiorespiratory support.

## Conclusion

Respiratory effort drives RSA in healthy humans, while the pulmonary stretch reflex contributes little to RSA. We used NIV to maintain the pulmonary stretch during the respiratory phases and reduce the central respiratory drive in healthy humans. The arterial baroreflex and cardiopulmonary reflexes have the potential to modify respiratory sinus arrhythmia, but they are not the main contributors.

## Data Availability

The raw data supporting the conclusions of this article will be made available by the authors, without undue reservation.
